# Supplementary material for: Neutralization activity in chronic HIV infection is characterized by a distinct programming of follicular helper CD4 T cells
Source: bioRxiv. 2024 Aug 3:2024.07.31.605954. Preprint. [Version 1] doi: 10.1101/2024.07.31.605954 (PMC11312598; doi:10.1101/2024.07.31.605954)
Supplement: Supplement 1 [file NIHPP2024.07.31.605954v1-supplement-1.pdf]

bioRxiv preprint doi: https://doi.org/10.1101/2024.07.31.605954; this version posted August 3, 2024. The copyright holder for this preprint (which was not certified by peer review) is the author/funder. This article is a US Government work. It is not subject to copyright under 17 USC 105 and is also made available for use under a CC0 license.

Supplementary Materials

Materials and Methods

Fig.S1: Cross-neutralization profiles of NN and N study participants, and heterogeneity of B-cell follicle shape.

Fig.S2: Phenotypic characterization of TFH cells.

Fig.S3: *In situ* characterization of TFH cells.

Fig.S4: Identification of CD4 T cell subsets using sc-RNA analysis of LMNCs.

Fig.S5: Regulatory T cell HistoCytometry gating strategy and Entropy-H(x) plots

Fig.S6: Identification of B cell clusters using sc-RNA analysis of LMNCs.

Table S1: Clinical Information of study participants

Table S2: Study Assays

Table S3: Flow Cytometry Antibodies

Table S4: Multiparameter Imaging Antibodies
